# FHL-1 is not involved in pressure overload-induced maladaptive right ventricular remodeling and dysfunction

**DOI:** 10.1007/s00395-019-0767-5

**Published:** 2020-01-24

**Authors:** Christine Veith, Dariusch Neghabian, Himal Luitel, Jochen Wilhelm, Bakytbek Egemnazarov, Caja Muntanjohl, Jan-Hendrik Fischer, Bhola Kumar Dahal, Ralph Theo Schermuly, Hossein Ardeschir Ghofrani, Friedrich Grimminger, Ludger Fink, Grazyna Kwapiszewska, Norbert Weissmann, Akylbek Sydykov

**Affiliations:** 1 Member of the German Center for Lung Research (DZL), Excellence Cluster Cardio-Pulmonary System (ECCPS), Universities of Giessen and Marburg Lung Center (UGMLC), Justus-Liebig-University Giessen, Aulweg 130, 35392 Giessen, Germany; 2 Ludwig Boltzmann Institute for Lung Vascular Research, Medical University of Graz, Neue Stiftingtalstrasse 6/VI, 8010 Graz, Austria

**Keywords:** Right ventricular hypertrophy, Pulmonary arterial banding, Pressure overload, Four and-a-half LIM domain 1 protein, Cytoskeletal proteins

## Abstract

**Aims:**

The cytoskeletal signaling protein four and-a-half LIM domains 1 (FHL-1) has recently been identified as a novel key player in pulmonary hypertension as well as in left heart diseases. In this regard, FHL-1 has been implicated in dysregulated hypertrophic signaling in pulmonary arterial smooth muscle cells leading to pulmonary hypertension. In mice, FHL-1-deficiency (FHL-1^−/−^) led to an attenuated hypertrophic signaling associated with a blunted hypertrophic response of the pressure-overloaded left ventricle (LV). However, the role of FHL-1 in right heart hypertrophy has not yet been addressed.

**Methods and results:**

We investigated FHL-1 expression in C57Bl/6 mice subjected to chronic biomechanical stress and found it to be enhanced in the right ventricle (RV). Next, we subjected FHL-1^−/−^ and corresponding wild-type mice to pressure overload of the RV by pulmonary arterial banding for various time points. However, in contrast to the previously published study in LV-pressure overload, which was confirmed here, RV hypertrophy and hypertrophic signaling was not diminished in FHL-1^−/−^ mice. In detail, right ventricular pressure overload led to hypertrophy, dilatation and fibrosis of the RV from both FHL-1^−/−^ and wild-type mice. RV remodeling was associated with impaired RV function as evidenced by reduced tricuspid annular plane systolic excursion. Additionally, PAB induced upregulation of natriuretic peptides and slight downregulation of phospholamban and ryanodine receptor 2 in the RV. However, there was no difference between genotypes in the degree of expression change.

**Conclusion:**

FHL-1 pathway is not involved in the control of adverse remodeling in the pressure overloaded RV.

## Introduction

Pulmonary hypertension (PH) is a life threatening disease, characterized by an increase in pulmonary vascular resistance and pulmonary arterial pressure [8, 24]. In this regard, the right ventricle (RV) is exposed to chronic pressure overload. Moreover, the outcome of patients with PH is strongly determined by the response of the RV to the increased afterload [6, 41]. At the onset, the RV adapts to the increased afterload and biomechanical stress by compensatory processes, including cardiomyocyte hypertrophy, elevated capillary density and synthesis of extracellular matrix proteins [6, 12, 35]. At a certain time point, hypertrophy is no longer compensated and right ventricular failure, characterized by contractile dysfunction, extensive fibrosis, chamber dilatation and capillary rarefaction occurs, leading eventually to death [41].

Until now, the cellular and molecular mechanisms, underlying the transition from compensated hypertrophy to heart failure have largely been neglected. Little is known about how biomechanical stress is sensed by the cardiomyocyte sarcomere and then transduced into dysregulated molecular and cellular mechanisms underlying the disease pathogenesis [6, 46].

Two general models have been suggested to explain how cells transduce physical into molecular signals, which then induce alterations in gene expression: (1) via mechanosensitive ion channels, facilitating ion fluxes in response to mechanical cues and activating signaling cascades [29]; (2) via altered cell adhesion sites (focal adhesions, also called costameres in cardiomyocytes) and actin cytoskeleton activating signaling cascades [5, 6, 22].

In this regard, the LIM domain containing focal adhesion protein four and-a-half LIM domains 1 (FHL-1) was identified as a part of a complex in the cardiomyocyte sarcomer that senses biomechanical stress-induced responses, important for the development of pathological left ventricular (LV) hypertrophy [37]. FHL-1 is predominantly expressed in skeletal muscle, heart [30], and pulmonary vasculature [26]. Its expression is elevated in idiopathic pulmonary arterial hypertension patients, in patients with LV hypertrophy [21] and in animal models of left heart hypertrophy [10, 17]. It has been shown that FHL-1 is essential for biomechanical stress sensing in the LV, since FHL-1 deficient mice exhibited an attenuated hypertrophic signaling, blunted hypertrophic response and preserved LV function after transverse aortic constriction (TAC) for 1 and 5 weeks [37].

In the present study, we hypothesized that molecular mechanisms underlying adaptation to biomechanical stress, at least in part, differ between RV and LV. Thus, we aimed to investigate if a loss of FHL-1, in contrast to the LV would not diminish hypertrophic signaling in the pressure-overloaded RV. Biomechanical stress to the RV was induced in FHL-1-deficient mice and their wild-type littermates by creating chronic pressure overload via pulmonary arterial banding (PAB).

## Methods

### Experimental design

To assess the in vivo impact of FHL-1, regulation of FHL-1 was assessed in right ventricular tissue homogenate samples from C57/BL6 mice subjected to either PAB or sham surgery for 1 week, 2 weeks, and 3 weeks. Samples were obtained from a previous study [28].

To assess the impact of FHL-1 on pressure overload-induced right ventricular remodeling, 8- to10-week-old male FHL-1 knockout mice (Crl:NIHBL(S)-FHL1tm; FHL-1^−/−^) and their littermates (wild-type; WT; Swiss) were subjected to either PAB or sham surgery for 3 days, 1 week, 3 weeks or 5 weeks. To assess the impact of FHL-1 on pressure overload-induced left ventricular remodeling, 8- to 10-week-old male FHL-1^−/−^ mice and their littermates were subjected to transverse aortic constriction (TAC) for 5 weeks. All animals were maintained under appropriate barrier conditions in a 12-h/12-h light–dark cycle and received standard laboratory food ad libitum and free access to water. All procedures involving animals were approved by the governmental Animal Ethics Committee (Regierungspraesidium Giessen) and have, therefore, been performed in accordance with the ethical standards laid down in the 1964 Declaration of Helsinki and its later amendments.

### Model of PAB

Right ventricular pressure overload was induced by PAB in FHL-1^−/−^ and WT Swiss mice as previously described [28]. Briefly, buprenorphine hydrochloride (Temgesic^®^, 0.1 mg/kg bw, Essex Pharma GmbH, Munich, Germany) was administered s.c. as an analgesic prior to operation. Surgery was performed under general anesthesia of 2% isoflurane (Forene^®^, Abbott, Wiesbach, Germany). The animals were placed on a heating pad to maintain body temperature and were ventilated with a rodent ventilator (MiniVent Type 845, Hugo Sachs Elektronik KG, March, Germany). After lateral thoracotomy to gain access to the pulmonary artery, the pulmonary artery was constricted using small titanium clips (Hemoclip^®^, Edward Weck, Morrisville, USA) and a modified, adjustable clip applier (Hemoclip^®^, Edward Weck). The chest cavity and skin was stitched by a 6-0 polypropylene suture (Prolene^®^, Ethicon, Norderstedt, Germany). In control mice, a sham operation without pulmonary artery occlusion was performed. Thereafter, animals were observed for various time periods post-surgery.

### Model of TAC

Left ventricular pressure overload was induced by TAC in FHL-1^−/−^ mice and their littermates as previously described [34]. Briefly, buprenorphine hydrochloride (Temgesic^®^, 0.1 mg/kg bw, Essex Pharma GmbH) was administered s.c. as an analgesic prior to operation. Surgery was performed under general anesthesia of 2% isoflurane (Forene^®^, Abbott). The animals were placed on a heating pad to maintain body temperature and were ventilated with a rodent ventilator (MiniVent Type 845, Hugo Sachs Elektronik KG). Small incision was made in the left second intercostal space. After identification of transverse aorta, a small titanium clip (Hemoclip^®^, Edward Weck) was placed around transverse aorta between innominate artery and left common carotid artery to make a constriction, restricting the lumen to a diameter of 0.3 mm. The chest cavity and skin was stitched by a 6-0 polypropylene suture (Prolene^®^, Ethicon).

### Echocardiography

Echocardiographic studies were performed at baseline and 1 day before final hemodynamic measurements. Mice were anesthetized using isoflurane (1.5% v/v) and placed on a controlled heating table and the core temperature, measured via rectal probe, was maintained at 37 °C. Echocardiographic images were acquired using a Vevo 770 high-resolution imaging system equipped with a 30-MHz RMV-707B scanning head (VisualSonics, Toronto, Canada). In PAB mice, the RV free wall thickness (RVWT) was measured in the right parasternal long-axis view and the RV internal diameter (RVID) was measured from the apical four-chamber view as maximal transverse diameter in the middle third of the RV during end-diastole. For assessment of the RV performance, the tricuspid annular plane systolic excursion (TAPSE) was measured. Images were then analyzed off-line by a single observer blinded to the respective treatments of mice.

In TAC mice, two-dimensional-guided M-mode images were recorded in the parasternal long-axis view. The average of three consecutive cycles was taken for analysis. Calculations were performed offline with the software Vevo LAB. The following parameters were derived: LV internal diameter at end-diastole (LVIDd), LV internal diameter at end-systole (LVIDs), end-diastolic interventricular septum thickness (IVSd) and left ventricular posterior wall thickness at end-diastole (LVPWd). Fractional shortening (LV FS) of the left ventricle was calculated as (LVIDd − LVIDs)/LVIDd × 100.

### Hemodynamic measurements

In vivo hemodynamic measurements were performed in separate groups at various time points after PAB (3 days, 1 week, 3 weeks, 5 weeks). Mice were anesthetized using isoflurane (1.5% v/v) and placed on controlled heating table and the core temperature, measured via rectal probe, was maintained at 37 °C. The right jugular vein was used for catheterization of the RV to measure right ventricular systolic pressure (RVSP), RV end-diastolic pressure (RVEDP), maximum rate of pressure change in the ventricle (max dP/dT), minimum rate of pressure change in the ventricle (min dP/dT) and time constant of isovolumic pressure decay Tau. Systemic arterial pressure (SAP) and mean arterial pressure (MAP) were measured by catheterizing the right carotid artery. Hemodynamic measurements were performed using a high-fidelity 1.4F micromanometer catheter (Millar Instruments, Houston, USA). Data were collected and analyzed with the PowerLab data acquisition system (MPVS-Ultra Single Segment Foundation system, AD instruments, Spechbach, Germany) and Labchart 7 software. Contractility index was calculated by the LabChart software as max dP/dT divided by the pressure at the time of max dP/dT. At the conclusion of the measurements, blood samples were obtained and the hearts were harvested.

### Tissue processing and histology

The ventricles were dissected free of the great vessels and atria. The RV was isolated from the left ventricle by dissection along the septal insertion and then was patted dry and weighed. The RV hypertrophy was assessed using the RV mass to tibia length (TL) ratio (RV/TL). LV hypertrophy was determined using the LV mass plus septum (LV + S) to TL ratio.

Freshly dissected RV tissue was fixed in 4% paraformaldehyde overnight, then dehydrated and embedded in paraffin, and sectioned at a thickness of 3 µm. To detect collagen fibers, RV sections were stained with 0.1% sirius red (sirius red F3B, Niepoetter, Bürstadt, Germany) in picric acid (Fluka, Neu-Ulm, Germany). For cardiomyocyte size determination transverse sections of the RV were stained with FITC-conjugated wheat germ agglutinin (WGA-FITC, Sigma Aldrich, Steinheim, Germany). For quantification of the capillaries, sections were stained with IB4-TRITC (Sigma Aldrich). Nuclei were stained with 4′,6-diamidino-2-phenylindole (DAPI, Invitrogen, Karlsruhe, Germany) and mounted in Flouro Care Anti-Fade Mountant (Biocare Medical, Berlin, Germany). Sections without WGA-FITC, IB4-TRITC, and DAPI staining were used as a negative control. Photomicrographs were quantified to determine the mean cross-sectional area of cardiomyocytes and interstitial collagen fraction using Leica Qwin V3 computer-assisted image analysis software (Leica Microsystem, Wetzlar, Germany). Photomicrographs were quantified to count the number of cardiomyocytes and capillaries using STEPanizer image analysis tool (University of Bern, Department of Anatomy, Bern, Switzerland). Myocardial capillary density was expressed as a number of capillaries per cardiomyocyte. Average data reflect results from at least four or five different hearts in each group (more than 100 cells for each heart).

### Immunohistochemical staining

Sections were deparaffinized in xylene for 3 × 10 min and rehydrated in 100% ethanol for 2 × 5 min, 95% ethanol for 1 × 5 min, 70% ethanol for 1 × 5 min, and (Phosphate Buffered Saline) PBS for 2 × 5 min. Antigen retrieval was performed with 0.05% trypsin (Zymed Laboratories, Invitrogen) for 10 min at 37 °C. Tissue was blocked for 1 h with 10% BSA before incubation with goat anti-FHL-1 (Abcam, Cambridge, UK; ab23937; 1:250 diluted) or rabbit anti-PCNA (Santa Cruz Biotechnology Inc., Heidelberg, Germany; sc7907; 1:100 diluted) antibodies followed by ImmPRESS anti-Goat or anti-Rabbit Ig (peroxidase) Polymer Detection Kit (Vector Laboratories, Burlingame, CA). Negative control experiments were performed with an isotype control (matched host species and isotype) antibody.

### Immunofluorescence staining

Sections were deparaffinized in xylene for 3 × 10 min and rehydrated in 100% ethanol for 2 × 5 min, 95% ethanol for 1 × 5 min, 70% ethanol for 1 × 5 min, and PBS for 2 × 5 min. Antigen retrieval was performed with 0.05% trypsin (Zymed Laboratories Inc., Invitrogen) for 10 min at 37 °C. Tissue was blocked for 1 h with 10% BSA prior to goat anti-FHL-1 (Abcam; ab23937; 1:100 diluted) or mouse anti-α-actinin (Sigma Aldrich; A5044; 1:100 diluted) antibody incubation overnight. After washing 4 × 5 min with 0.1% BSA in PBS, sections were incubated with Alexa Fluor^®^ Dye 488 and Alexa Fluor^®^ Dye 555 (Invitrogen; 488 goat, A21467; 555 mouse, A21427) antibodies for 1 h. Tissue was fixed for 10 min with 4% PFA and mounted with fluorescence Vectashield mounting medium (Vector Laboratories) including DAPI. Staining specificity was assessed via simultaneous staining of control sections with an isotype control antibody. For microscopic inspection, a Leica DMR microscope (Leica Microsystems) equipped with the following filters: AL 380–420 nm (DAPI), I3 420–512 nm (Alexa Fluor^®^ Dye 488), and N 2.1. 500–590 nm (Alexa Fluor^®^ Dye 555) (all from Leica) was used.

### RNA isolation, cDNA synthesis, and real-time PCR

RNA from C57/BL6 mice was isolated from snap-frozen right ventricles using the RNeasy Mini Kit (Qiagen, Hilden, Germany). RNA from FHL-1^−/−^ and WT Swiss mice was extracted from formalin-fixed paraffin-embedded heart tissue using the deparaffinization solution (Qiagen) and the RNeasy FFPE Kit (Qiagen). For cDNA synthesis the iScript™ cDNA Synthesis Kit (Bio-Rad Laboratories, Inc., Dreieich, Germany) was used. Real-time PCR was performed in a CFX Connect™ Real-Time PCR Detection System (Bio-Rad Laboratories, Inc.). The PCR reactions were set up using iTaq™ Universal SYBR^®^ Green Supermix (Bio-Rad). The following cycling conditions were chosen: 3 min at 95 °C, [5 s (sec) at 95 °C, 10 s at 59 °C, and 10 s at 72 °C] × 40. As the SYBR^®^Green I dye can bind non-selectively to the double-stranded DNA, melting curve analysis and gel electrophoresis were performed to confirm the exclusive amplification of the expected PCR product. The ΔCt values for each target gene were calculated by ΔCt = Ct reference gene—Ct target gene. Primer sequences are given from (5′–3′): mouse B2m (β2-microglobulin; FP: ATG CTA TCC AGA AAA CCC CTC A; RP: GCA GTT CAG TAT GTT CGG CT), mouse Anp (atrial natriuretic peptide; FP: GCT TCC AGG CCA TAT TGG AG; RP: GTC TAG CAG GTT CTT GAA ATC CA), mouse Bnp (brain natriuretic peptide; FP: TAT CTC AAG CTG CTT TGG GC; RP: ACA ACT TCA GTG CGT TAC AGC), mouse Atp2a2 (sarco(endo)plasmic reticulum calcium-ATPase 2; FP: GCC TTT GTA GAG CCG TTT GT; RP: TTT CTT TCC TGC CAC ACA CC), mouse Plb (phospholamban; FP: ATA CAG CTT CAT GCT CTG CAC; RP: TCT TCA CCT GCT TCT GTC TTG), mouse Ryr2 (ryanodine receptor 2; FP: CGA GCG TGT CCT GGG TAT AG; RP: TTG AGG ATG TTC CAC CAG GC), mouse αMhc (α myosin heavy chain; FP: TGT GGT GCC TCG TTC CA; RP: TTT CGG AGG TAC TGG GCT G), mouse βMhc (β myosin heavy chain; FP: GCA TTC TCC TGC TGT TTC CTT; RP: TGG ATT CTC AAA CGT GTC TAG TGA), mouse collagen 1a1 (FP: CTG ACG CAT GGC CAA GAA GA; RP: TAC CTC GGG TTT CCA CGT CT), mouse collagen 1a2 (FP: GCT TGC AGT AAC TTC GTG CCT; RP: CAG TGG GGC CCT TTC GTA CT), mouse collagen 3a1 (FP: AAA ACC CTG CTC GGA ACT G; RP: CTT GCA GCC TTG GTT AGG AT), mouse FHL-2 (FP: GGA AGG GCT CTG ACC TCT AAC A; RP: CAT TGC AGT GGT GGC AGT CAA), and mouse FHL-3 (FP: CCT GGC CCA CAG GTA GGA; RP: CGG TGC CCA GTG AGC C). The primers were intron spanning. B2m served as a reference gene.

### Western blotting

For protein extraction, murine heart samples were disrupted by grinding in liquid nitrogen and lysed with 150 µl NP-40 lysis buffer containing 20 mM Tris (pH 7.5), 150 mM NaCl, 1 mM EDTA (pH 8.0), 1 mM EGTA (pH 8.0), 0.5% NP-40, 2 mM Na3VO4 (pH 10) and 1 × Complete (Roche Diagnostics, Mannheim, Germany). After incubation on ice for 30 min, samples were centrifuged (20,000×*g*, 15 min, 4 °C). Protein concentrations were determined by a spectrophotometric assay (BCA assay, Pierce, Rockford, IL, USA). 20 μg/μL of protein extract was used for Western blotting. Protein samples were run on a 12% SDS polyacrylamide gel, followed by electrotransfer to a 0.45-μm PVDF membrane (ImmobilonTM-P, Millipore Corporation, Billerica, MA, USA). After blocking with 5% non-fat dry milk in PBS-T buffer (PBS with 0.1% Tween20) the membrane was incubated overnight at 4 °C with one of the following antibodies: anti-goat FHL-1 (Abcam, ab23937; 1:1000 diluted), anti-mouse α-tubulin (Santa Cruz Biotechnology Inc., sc5286; 1:1000 diluted); anti phospho-Akt (serine 473; #92715); anti-Akt (#9272); anti phospho-Erk1/2 (threonine 202/tyrosine 204; #9101) and anti-Erk1/2 (#9102; all from Cell Signaling, Boston, USA, all raised in rabbits and 1:1000 diluted). After washing 3× for 10 min with PBS-T buffer the membrane was incubated for 1 h with horseradish-peroxidase-labeled secondary antibodies (Promega, Madison, USA; anti-rabbit, W4021 and anti-mouse, W4011; Santa Cruz Biotechnology Inc., anti-goat, sc2020; all 1:5000 diluted). Afterwards, the membranes were washed 3× for 15 min. Final detection of proteins was performed using the Amersham ECL Plus Western Blotting Detection System (GE Healthcare, Munich, Germany). Antibodies were removed by incubating the membrane for 30 min with stripping buffer containing 10 mL H_2_O, 5 mL 1 M glycine, and 750 μL 25% HCl.

### Statistical analysis

Data are presented as mean ± standard error of mean (SEM). Differences between more than two groups were assessed by one- or two-way ANOVA followed by Dunnett’s or Tukey’s multiple comparisons post hoc tests. A *p* value less than 0.05 was considered significant for all analyses. Statistical analysis was performed using Prism 7 (GraphPad Software Inc., San Diego, USA). *n* numbers are indicated in or below the respective graphs.

## Results

### FHL-1 expression due to chronic pressure overload

**Fig. 1:**
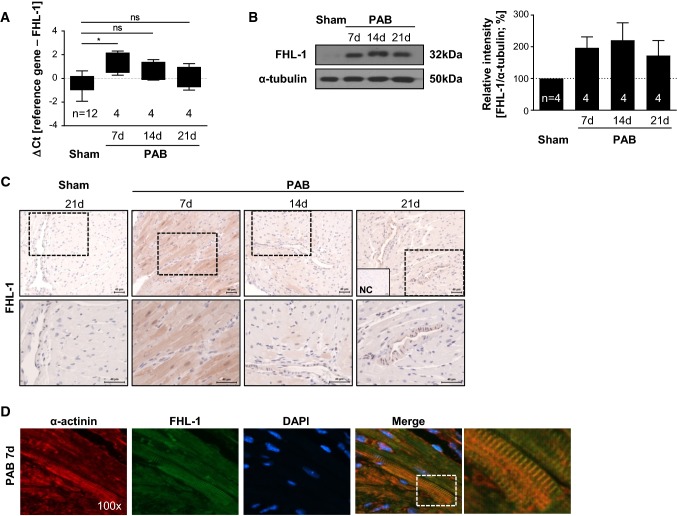
Changes in FHL-1 expression after PAB. **a** Real-time PCR analysis of FHL-1 expression in right ventricles of C57/BL6 mice after sham or PAB for 7, 14 or 21 days (d). Data were analyzed by analysis of variance followed by Dunnett’s multiple-to-one comparison post hoc tests and are presented as mean ± standard error of mean (SEM). *Significant differences between sham and PAB; *ns* not significantly different. **b** Left: representative Western blot analysis of FHL-1 expression in right ventricles of C57/BL6 mice after sham or PAB. Right: densitometric analysis. Data were normalized to β-tubulin and sham was set to 100%. Data were analyzed by analysis of variance followed by Dunnett’s multiple-to-one comparison post hoc tests and are presented as mean ± SEM. **c** Immunohistochemical staining of FHL-1 (in red) after sham or PAB in C57/BL6 mice. *NC* isotype control staining. **d** Immunofluorescence staining of α-actinin (in red) and FHL-1 (in green) after 7 days of PAB in C57/BL6 mice. Nuclear staining was performed with DAPI (blue)

In a previous study, FHL-1 was identified as a key protein in a biomechanical stress sensing complex in the left heart as FHL-1 deficient mice exhibited an attenuated hypertrophic signal transduction and preserved LV function after TAC [37]. To decipher the role of FHL-1 in hypertrophic signaling leading to right heart hypertrophy, we first assessed the FHL-1 expression in an in vivo model of pressure overload-induced right heart hypertrophy caused by PAB in C57/BL6 mice [28]. Pressure overload led to an increase in FHL-1 mRNA expression in the RV. The time course revealed a peak in expression after 7 days of PAB (Fig. 1a). FHL-1 protein expression was also induced by PAB (Fig. 1b). Immunohistochemistry confirmed highly elevated FHL-1 levels following 7 days of PAB with strong immunoreactivity in cardiomyocytes (Fig. 1c). Immunofluorescence staining revealed co-localization of FHL-1 and α-actinin (Fig. 1d), a microfilament protein, necessary for attachment of actin filaments to Z-disks in cardiac muscles [18].

### Hypertrophic signaling following PAB

**Fig. 2:**
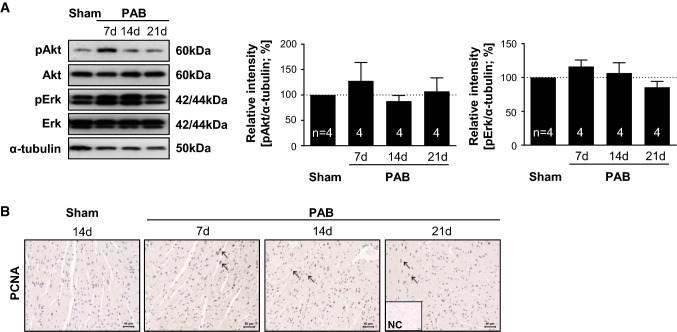
Hypertrophic signaling following PAB. **a** Left: representative Western blot analysis of Akt and Erk phosphorylation in right ventricles of C57/BL6 mice after sham or PAB for 7, 14 or 21 days (d). Right: densitometric analysis of Akt and Erk phosphorylation. Data were normalized to β-tubulin and sham was set to 100%. Data were analyzed by analysis of variance followed by Dunnett’s multiple-to-one comparison post hoc tests and are presented as mean ± standard error of mean (SEM). **b** Immunohistochemical staining of PCNA (in red) in C57/BL6 mice after sham or PAB. *NC* isotype control staining. Arrows indicate PCNA-positive nuclei

It has previously been demonstrated that TAC leads to induction of hypertrophic signaling in the LV [37]. Thus, we sought to determine whether PAB can also induce hypertrophic signaling in the RV of C57/BL6 mice. Western blot analysis demonstrated no prominent changes in Akt and Erk phosphorylation, two MAPK components, following PAB (Fig. 2a). Immunohistochemistry showed elevated PCNA levels, as well as nuclear localization after PAB (Fig. 2b).

### Structural and functional changes after pressure overload-induced RV hypertrophy in FHL-1^−/−^ mice

**Fig. 3:**
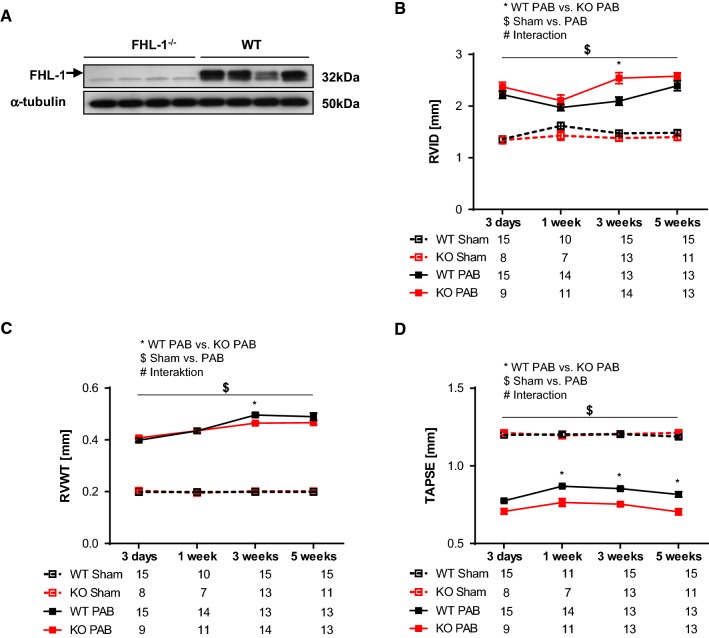
PAB-induced echocardiographic changes in WT Swiss and FHL-1^−/−^ mice. **a** Western blot analysis of FHL-1 expression in right ventricles derived from WT Swiss and FHL-1^−/−^ mice. **b** Right ventricular internal diameter (RVID) after sham or PAB for 3 days (d) or 1, 3 or 5 weeks in WT and FHL-1^−/−^ mice. **c** Right ventricular wall thickness (RVWT). **d** Tricuspid annular plane systolic excursion (TAPSE). *Significant differences between WT PAB and FHL-1^−/−^ PAB. ^$^Significant differences between sham and PAB. ^#^Interaction. Data were analyzed by 2-way analysis of variance followed by Tukey’s multiple comparison post hoc tests and are presented as mean ± standard error of mean (SEM)

It has previously been shown that FHL-1^−/−^ mice exhibit a blunted and beneficial response to left ventricular pressure overload induced by TAC [37]. In order to reassure that knockout mice used in our PAB study do not differ in their response to pressure overload from those in the previous study published a decade ago, we subjected FHL-1^−/−^ mice and their littermates to TAC for 5 weeks. In detail, FHL-1^−/−^ mice had a significantly smaller increase in (LV + S)/TL ratio compared to WT mice following 5 weeks of TAC (Table 1). Moreover, LVPWd and IVSd were significantly smaller in FHL-1^−/−^ hearts compared to controls (Table 1). Importantly, FHL-1^−/−^ mice displayed a better preserved LV function following TAC compared to WT mice (Table 1). Furthermore, absence of FHL-1 protein expression in RV derived from FHL-1^−/−^ mice was proven by Western blotting (Fig. 3a).

**Table 1:** In vivo echocardiographic assessment of cardiac size and function in WT and FHL-1^−/−^ mice following 5 weeks of TAC

	WT (*n *= 10)	Fhl-1^−/−^ (*n *= 6)
BW (g)	32.19 ± 0.63	30.68 ± 1.18
HR (bpm)	535.1 ± 8.0	574.2 ± 7.7*
(LV+S)/TL	6.0 ± 0.14	5.03 ± 0.14*
IVSd (mm)	0.94 ± 0.02	0.82 ± 0.02*
LVIDd (mm)	4.01 ± 0.10	3.76 ± 0.10
LVIDs (mm)	2.80 ± 0.12	2.34 ± 0.05*
LVPWd (mm)	0.92 ± 0.02	0.82 ± 0.02*
LV FS	30.72 ± 1.11	38.07 ± 0.78*

Data were analyzed by 2-way analysis of variance followed by Tukey’s multiple comparison post hoc tests and are presented as mean ± standard error of mean (SEM)

*BW* body weight, *HR* heart rate, *(LV + S)/TL* (left ventricle + septum)/tibia length, *LVSd* end-diastolic interventricular septum thickness, *LVIDd* LV internal diameter at end-diastole, *LVIDs* LV internal diameter at end-systole, *LVPWd* LV posterior wall thickness at end-diastole, *LV FS* left ventricular fractional shortening

*Significant differences. WT, *n *= 10; FHL-1^−/−^, *n *= 6

In the present study, we aimed to investigate the effects of FHL-1 ablation in an in vivo model of chronic pressure overload-induced RV hypertrophy. We hypothesized that FHL-1, at least in part, plays different roles in the RV and LV hypertrophy. Biomechanical stress was induced in FHL-1^−/−^ and WT mice by PAB. Following PAB, echocardiography was performed to evaluate RV remodeling and function. RVID (Fig. 3b) and RVWT (Fig. 3c) were increased, whereas TAPSE (Fig. 3d) was decreased after PAB at all time-points investigated compared to sham mice. RV remodeling in FHL-1^−/−^ mice after 3 weeks of PAB was characterized by more pronounced dilatation of the RV compared to WT PAB mice, as evidenced by a larger RVID and a smaller RVWT (Fig. 3b, c). TAPSE decreased 3 days after PAB and remained depressed thereafter compared to sham mice (Fig. 3d). Interestingly, FHL-1^−/−^ mice displayed significantly lower values of TAPSE compared to those in WT PAB mice after 1, 3, and 5 weeks of PAB (Fig. 3d).

**Fig. 4:**
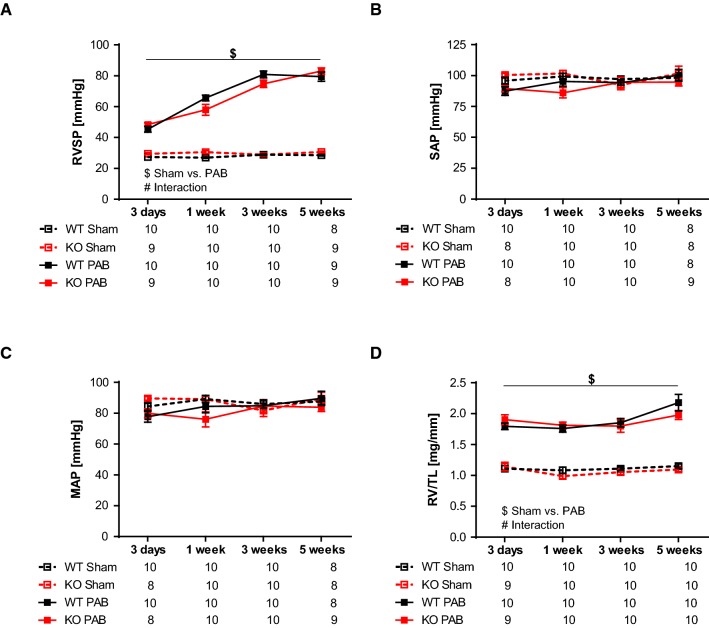
PAB-induced hemodynamic and right ventricular morphological changes in WT Swiss and FHL-1^−/−^ mice. **a** Right ventricular systolic pressure (RVSP) after sham or PAB for 3 days (d) or 1, 3 or 5 weeks in WT and FHL-1^−/−^ mice. **b** Systolic arterial pressure (SAP). **c** Mean arterial pressure (MAP). **d** Ratio of right ventricle to tibia length. ^$^Significant differences between sham and PAB. ^#^Interaction. Data were analyzed by 2-way analysis of variance followed by Tukey’s multiple comparison post hoc tests and are presented as mean ± standard error of mean (SEM)

**Fig. 5:**
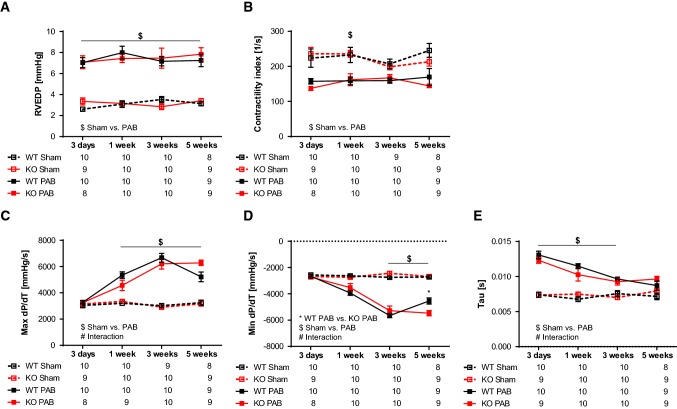
PAB-induced hemodynamic changes in WT Swiss and FHL-1^−/−^ mice. **a** Right ventricular end-diastolic pressure (RVEDP) after sham or PAB for 3 days (d) or 1, 3 or 5 weeks in WT and FHL-1^−/−^ mice. **b** Contractility index. **c** Maximal changes in blood pressure during isovolumetric contraction (max dP/dT). **d** Minimal changes in blood pressure (min dP/dT). **e** Tau, time constant of RV relaxation. *Significant differences between WT PAB and FHL-1^−/−^ PAB. ^$^Significant differences between sham and PAB. ^#^Interaction. Data were analyzed by 2-way analysis of variance followed by Tukey’s multiple comparison post hoc tests and are presented as mean ± standard error of mean (SEM)

Right heart catheterization revealed a gradual increase in RVSP after PAB (Fig. 4a). RVSP values were not different between WT and FHL-1^−/−^ mice (Fig. 4a). SAP (Fig. 4b) and MAP (Fig. 4c) were affected neither by the type of surgery nor by genotype. The degree of RV hypertrophy, depicted by the ratio of the RV mass to tibia length, increased after PAB but again was not influenced by the FHL-1 ablation (Fig. 4d). RVEDP was higher in PAB than in sham mice (Fig. 5a), indicating worsened RV diastolic function in these mice. Contractility index, a parameter for systolic function, was decreased in PAB mice (Fig. 5b). Velocity of ventricular contraction (max dP/dT) was significantly enhanced starting after 1 week of PAB (Fig. 5c). Velocity of ventricular relaxation (min dP/dT) was significantly decreased in a time-dependent manner following PAB (Fig. 5d). Tau significantly increased following PAB but gradually decreased to sham level by 5 weeks (Fig. 5e). In the hemodynamic parameters, WT did not differ from FHL-1^−/−^ mice in their response to chronic pressure overload.

### RV remodeling in FHL-1^−/−^ mice in response to chronic pressure overload

**Fig. 6:**
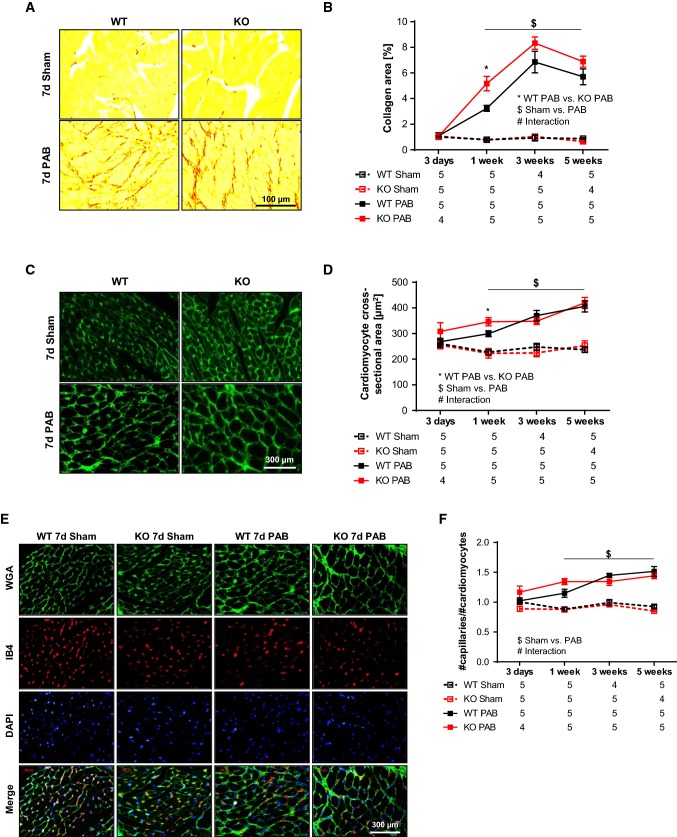
PAB-induced right ventricular remodeling in WT Swiss and FHL-1^−/−^ mice. **a** Representative pictures of sirius red in picric acid staining to detect fibrotic changes (in red) after sham or PAB for 7 days in WT and FHL-1^−/−^ mice. **b** Quantification of right ventricular fibrosis after sham or PAB for 3 days (d) or 1, 3 or 5 weeks in WT and FHL-1^−/−^ mice. **c** Representative pictures of FITC conjugated wheat germ agglutinin (WGA FITC; in green) staining to determine cell size. **d** Quantification of cardiomyocyte size depicted by cross-sectional area. **e** Representative pictures of FITC conjugated wheat germ agglutinin (WGA FITC; in green) and IB4-TRITC (in red) staining to determine cell size and number of capillaries. Nuclear staining was performed with DAPI (blue). **f** Quantification of number of capillaries to cardiomyocytes. *Significant differences between WT PAB and FHL-1^−/−^ PAB. ^$^Significant differences between sham and PAB. ^#^Interaction. Data were analyzed by 2-way analysis of variance followed by Tukey´s multiple comparison post hoc tests and are presented as mean ± standard error of mean (SEM)

Sirius red staining revealed an increase in fibrosis after PAB. Fibrosis was more prominent in FHL-1^−/−^ mice after 1 week of PAB compared to WT PAB mice (Fig. 6a, b). RV cardiomyocyte cross-sectional area increased in a time-dependent manner after PAB. Degree of cardiomyocyte hypertrophy was comparable between WT and FHL-1^−/−^ mice (Fig. 6c, d). Finally, myocardial capillary density was assessed, depicting a gradual increase in capillarization following PAB, but with no differences between genotypes (Fig. 6e, f).

### Molecular alterations in the RV following PAB in FHL-1^−/−^ mice

**Fig. 7:**
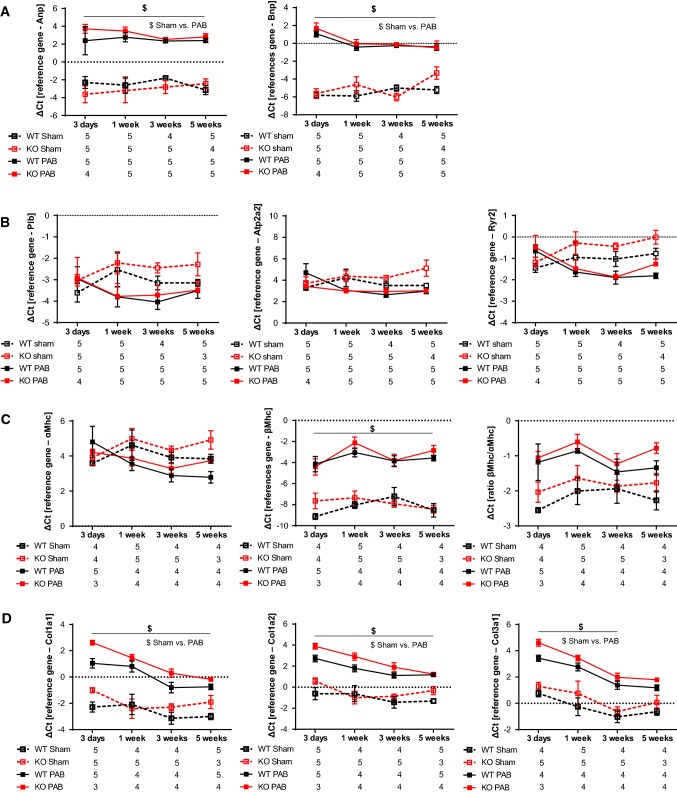

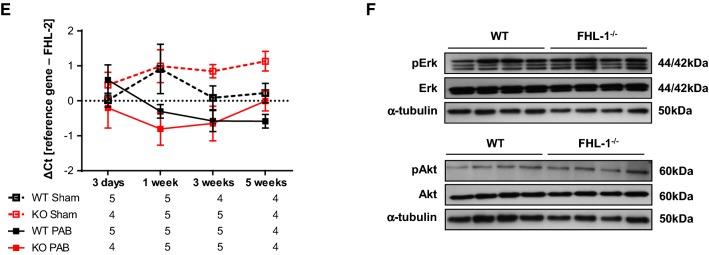
Changes in gene expression after PAB in WT Swiss and FHL-1^−/−^ mice. **a** Ventricular wall stress markers, left: Anp and right: Bnp after sham or PAB for 3 days (d) or 1, 3 or 5 weeks. *n* = 4–6 each. **b** Ca^2+^-handling proteins, left: Plb, middle: Atp2a2 and right: Ryr. *n* = 4–6 each. **c** Fetal genes, left: α-Mhc, middle: β-Mhc and right: β-Mhc/α-Mhc ratio. **d** Collagens, left: collagen 1a1, middle: collagen 1a2 and right: collagen 3a1. **e** FHL-2. ^$^Significant differences between sham and PAB. Data were analyzed by 2-way analysis of variance followed by Tukey´s multiple comparison post hoc tests and are presented as mean ± standard error of mean (SEM). **f** Western blot analysis of Erk and Akt phosphorylation in right ventricles of WT and FHL-1^−/−^ mice after 3 weeks of PAB

Next, we assessed how pressure overload affected right ventricular gene expression. We screened for the ventricular wall stress proteins Anp and Bnp, Ca^2+^-dependent proteins Plb, Atp2a2 and Ryr2, adult and fetal isoforms of myosin heavy chain (αMhc and βMhc) and finally collagens (collagen 1a1, collagen 1a2 and collagen 1a3). All pathways investigated reflect different aspects of heart adaptation and remodeling [14]. Quantitative realtime PCR analysis showed an increase in Anp and Bnp expression in RV tissues from PAB mice at all time-points investigated. Expression does not differ in WT and FHL-1^−/−^ mice (Fig. 7a). mRNA expression of Plb, Atp2a2 and Ryr2 trended to be decreased in PAB mice, however did not reach statistical significance. mRNA expression was not affected by the genotype (Fig. 7b). βMhc/αMhc ratio was markedly higher in PAB than in sham mice. Again, genotype did not influence gene expression (Fig. 7c). Finally, collagen 1a1, collagen 1a2, and collagen 3a1 mRNA expression was more prominent after chronic pressure overload, without any differences between genotypes (Fig. 7d). To rule out potential compensatory molecular mechanisms, expression of the FHL-1 family members FHL-2 and FHL-3 was assessed in RV form WT and FHL-1^−/−^ mice following sham/PAB, revealing no compensatory regulation for FHL-2 (Fig. 7e). FHL-3 expression in RV tissue was under detection level (data not shown).

Finally, we assessed how pressure overload affected RV hypertrophic signaling. In this regard, it was shown by Sheikh et al. [37] that FHL-1^–/–^ hearts showed a significant loss in Erk phosphorylation following TAC. In contrast, Erk and Akt activation was similar in WT and FHL-1^−/−^ RV following PAB (Fig. 7f).

## Discussion

The main cause of death of PAH patients is RV failure [8]. Maladaptive remodeling in response to chronic pressure overload could be the mechanism leading to heart failure and finally to death. Thus, understanding such mechanisms is important for development of novel therapies preventing RV failure in PH. In this regard, FHL-1 has been shown to be an essential molecule mediating maladaptive remodeling in LV pressure overload induced by TAC [37]. Moreover, previously, we and others have demonstrated the increase of FHL-1 in PH [1, 26].

Assuming that signaling pathways underlying adaptation mechanisms might, at least in part, differ between RV and LV, we aimed to investigate the role of FHL-1 in RV remodeling. First, we assessed the influence of the elevated RV afterload on the FHL-1 expression and finally the role of FHL-1 ablation in vivo in chronic pressure overload-induced right heart hypertrophy. Chronic pressure overload/PAB in C57/BL6 mice led to an increase in FHL-1 expression in the RV tissue, confirming microarray analysis from different groups [25, 39]. Similarly, induction of the FHL-1 expression in the LV tissue has been observed in animal models of left heart hypertrophy induced by TAC, as well as in patients with left heart diseases [10, 17, 19, 20, 25, 27, 47].

Immunofluorescence staining of the RV depicted localization of FHL-1 at the cardiomyocyte sarcomere, place of force transduction and contraction, as evidenced by the co-localization of FHL-1 and α-actinin. Thus, elevated FHL-1 expression following chronic pressure overload and localization at place of force transduction might point to a biomechanical stress sensor and transducer function/role of FHL-1 in the RV. In line with the previous publication [37], we observed a trend towards an activation of the Erk signaling in C57/BL6 mice, which is downstream to FHL-1.

Therefore, we subjected FHL-1^−/−^ mice to chronic pressure overload. PAB induced an increase in RVSP, leading to RV hypertrophy, dilatation and impaired function in WT mice. Maladaptive RV remodeling was evident from an enlargement in RVWT, significant chamber dilatation, cardiomyocyte hypertrophy and an increased fibrosis. All these mechanisms lead to RV dysfunction, as demonstrated by a reduced contractility index and TAPSE and elevated RVDEP. Similar observations were already made for C57/BL6 mice [14, 28].

In contrast to the LV as presented by others [37] and shown here, FHL-1 ablation did not blunt hypertrophic signaling and did not preserve RV function. Furthermore, neither expression of ventricular wall stress markers (Anp and Bnp), nor expression of Ca^2+^ handling proteins (Plb, Atp2a2 and Ryr2), collagens (collagen 1a1, collagen 1a2 and collagen 3a1), and myosin heavy chain (αMhc and βMhc) was affected by the genotype. In contrast, in vitro, in neonatal rat cardiomyocytes, FHL-1 overexpression induced Anp, Bnp, and βMhc mRNA expression [47], suggesting that the in vitro situation in cell culture might not necessarily reflect the in vivo situation in our experimental setup. However, neonatal cardiomyocytes used for in vitro studies are usually derived mostly from the left ventricle.

In general, we confirmed elevated expression of ventricular wall stress markers (Anp and Bnp) and βMhc after chronic pressure overload [13, 14]. However, we did not observe any differences between the genotypes. This suggests a comparable degree of RV wall stress between WT and FHL-1^−/−^ mice, also reflected by similar Anp and Bnp levels. A similar expression of Ca^2+^-handling proteins suggests a comparable degree of sarcomere and thus contractile function. Indeed, invasive hemodynamics measurements revealed comparable levels of contractility index changes between genotypes. In contrast to other studies, we used outbred Swiss mice, pointing that inter-stain differences [2, 3, 40] might have influenced the role of FHL-1 on cardiac function and gene expression. Thus, we cannot exclude that FHL-1 might play a more prominent role in the responses of the RV to pressure overload in mice with a different background or in other models of RV remodeling. In addition, intra-stain variations due to genetic heterogeneity within one strain [2] might have affected the role of FHL-1 on cardiac function and gene expression. However, this potential genetic drift was clearly disproven within the present study, since we confirmed the role of FHL-1 in maintenance of LV function in FHL-1^−/−^ mice, which was already published more than a decade ago [37].

Screening for compensatory protein expression in the RV following FHL-1 ablation revealed no changes in the expression of the FHL-1 family member FHL-2. Similar observations were made for the LV [37]. Moreover, FHL-2^–*/*–^ mice did not exhibit any significant differences in the hypertrophic response following TAC, when compared with WT controls [9]. Thus, other cytoskeletal and/or LIM proteins might have compensated the FHL-1 ablation in our PAB model.

We confirmed the induction of chronic pressure overload RV hypertrophy and fibrosis by PAB in outbred Swiss mice as already described for inbred C57BL/6 J mice and rats [13, 14, 25, 28]. However, in contrast to the LV, FHL-1^−/−^ mice did not show maintenance of the RV function and blunted hypertrophic response, indicating that pathways causing left heart maladaptation cannot always be transferred to the RV. The reason for the observed different responses to pressure overload of the RV and LV in FHL-1^−/−^ mice remains unclear. Importantly, RV and LV have different embryological origin [7, 32], structure, and function, suggesting that their adaptation mechanisms to biomechanical stress could be different [23, 25, 31, 42]. Indeed, rat LV and RV were shown to respond differently to continuous long-term norepinephrine infusion [23]. Norepinephrine induced hypertrophy in the LV while no hypertrophy occurred in the RV [4, 23]. Similar to our findings, differential hypertrophic responses in the two ventricles were reported in Sirtuin-3 knockout mice [44]. Sirtuin-3 knockout mice displayed augmented left ventricular hypertrophy in response to TAC [38]. On the contrary, when Sirtuin-3 knockout mice were exposed to chronic hypoxia, they developed PH and RV hypertrophy that was indistinguishable from those in wild-type littermates [44]. Several studies demonstrated that LV and RV differ in the expression profiles of genes and proteins, suggesting that these differences may generate specific molecular patterns with relevance to their pathophysiology [13, 15, 16, 33, 36, 43] and thus responses to therapies [4, 11, 23, 45]. We speculate that the responsiveness of FHL-1 to stress signals could be influenced by other proteins that differ between LV and RV. Alternatively, different independent signaling pathways might underlie pressure overload-induced remodeling in the LV and RV.

Thus, upregulation of FHL-1 in the RV following PAB suggests its contribution to the development of RV remodeling. However, no critical role for FHL-1 was found in pressure overload-induced maladaptive RV remodeling and dysfunction in mice. This observation suggests that, in contrast to the LV, FHL-1 is not involved during pathologic remodeling in the pressure overload-stressed RV.
